# Systematic assessment of template-based genome-scale metabolic models created with the BiGG Integration Tool

**DOI:** 10.1515/jib-2022-0014

**Published:** 2022-09-05

**Authors:** Alexandre Oliveira, Emanuel Cunha, Fernando Cruz, João Capela, João C. Sequeira, Marta Sampaio, Cláudia Sampaio, Oscar Dias

**Affiliations:** Centre of Biological Engineering, University of Minho, 4710-057 Braga, Portugal; LABBELS –Associate Laboratory, Braga, Guimarães, Portugal

**Keywords:** BiGG Integration Tool, BiGG models, genome-scale metabolic models, *merlin*

## Abstract

Genome-scale metabolic models (GEMs) are essential tools for *in silico* phenotype prediction and strain optimisation. The most straightforward GEMs reconstruction approach uses published models as templates to generate the initial draft, requiring further curation. Such an approach is used by BiGG Integration Tool (BIT), available for *merlin* users. This tool uses models from BiGG Models database as templates for the draft models. Moreover, BIT allows the selection between different template combinations. The main objective of this study is to assess the draft models generated using this tool and compare them BIT, comparing these to CarveMe models, both of which use the BiGG database, and curated models. For this, three organisms were selected, namely *Streptococcus thermophilus*, *Xylella fastidiosa* and *Mycobacterium tuberculosis.* The models’ variability was assessed using reactions and genes’ metabolic functions. This study concluded that models generated with BIT for each organism were differentiated, despite sharing a significant portion of metabolic functions. Furthermore, the template seems to influence the content of the models, though to a lower extent. When comparing each draft with curated models, BIT had better performances than CarveMe in all metrics. Hence, BIT can be considered a fast and reliable alternative for draft reconstruction for bacteria models.

## 1 Introduction

The reconstruction of metabolic networks in Systems Biology has peaked at an unprecedented scale, with over 6000 Genome-Scale Metabolic Models (GEMs) representing the metabolism of 434, 40 and 117 taxonomic families of bacteria, archaea and eukarya, respectively [1]. GEMs can predict the metabolic behaviour of an organism in several genetic and environmental conditions using methods, such as Flux Balance Analysis (FBA) [2]. Therefore, GEMs are relevant computational biology tools due to their many metabolic engineering applications, such as *in silico* strain optimisation [3].

Despite the comprehensive collection of GEMs, only a small share of these organisms (113 bacteria, ten archaea, and 60 eukaryotes) have manually and high-quality GEM reconstructions [1]. As of 2010, the Biochemical, Genetic and Genomic (BiGG) Models database has collected a wide variety of these high-quality and manually curated GEMs [4]. Up to the present date, this knowledgebase integrates 108 GEM reconstructions from bacteria, archaea and eukarya organisms. Moreover, BiGG Models provides the standardisation and unification of the reactions and metabolites available in these GEMs using universal non-redundant identifiers called BiGG identifiers. Then, reactions, metabolites and genes are mapped to external databases such as Kyoto Encyclopedia of Genes and Genomes (KEGG) [5], BioCyc [6], National Center for Biotechnology Information (NCBI) protein [7], among others.

The reconstruction of GEMs can usually follow two diverse approaches: bottom-up [8] and top-down [9].

The fast and automated reconstruction of metabolic networks consists of reconstructing a universal simulation-ready model that can be carved into an organism-specific model by pruning metabolic reactions for which genomic evidence is missing. CarveMe computational tool is an example of this top-down approach, used to create microbial community models by merging fast and automated single-species GEMs [9].

On the other hand, the widely-used bottom-up approach comprehends four main steps for generating high-quality metabolic networks [8]. This approach includes the genome functional annotation, network refinement and gap-filling, conversion to a stoichiometric model, and validation with experimental data. Although the bottom-up paradigm provides a protocol for reconstructing high-quality and relevant GEMs, the process tends to be time-consuming. Thus, fast and automated reconstructions have gained more interest in recent years.

With this in mind, we propose a novel computational tool for the fast reconstruction of GEMs using the BiGG Models database. The BiGG Integration Tool (BIT) has been implemented in the Metabolic Models Reconstruction Using Genome-Scale Information (*merlin*) software [10, 11]. This tool only requires the organism’s genome sequence to perform homology searches against the BiGG Models database and assemble a draft metabolic network. Thus, one can easily generate draft metabolic networks using only the Graphical User Interface (GUI).

In this study, we have used BIT to create several draft metabolic networks for three bacteria, namely *Streptococcus thermophilus*, *Xylella fastidiosa* and *Mycobacterium tuberculosis*. Gram negative and Gram positive bacteria available in the BiGG database were selected as case studies for this work [12–14]. Then, the metabolic content of the draft models was compared with reference GEMs [15–17]. Moreover, CarveMe’s top-down approach [9] has also been used to generate draft GEMs that were equally assessed against the BIT and reference reconstructions.

## 2 Workflow

BIT is a fully automated tool to generate draft GEMs using the BiGG Models database. This computational tool has been implemented in *merlin*, which is a graphical and user-oriented solution for the reconstruction of GEMs [10, 11].

BIT takes the genome sequence of the organism of interest and parameters related to the BLAST homology search (scoring matrix, e-value, and bit score) and gene-protein-reaction (GPR) associations.

**Figure 1:**
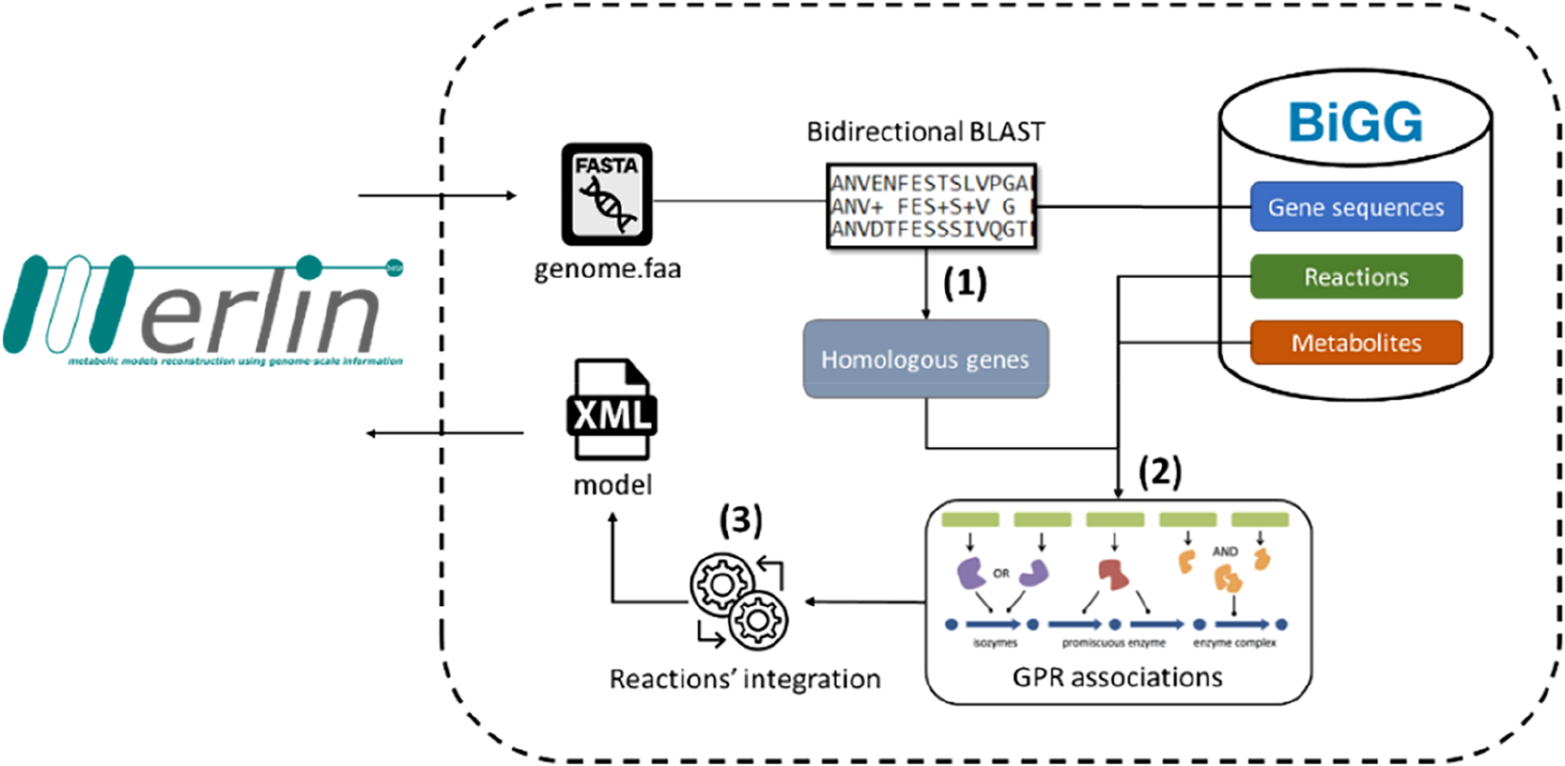
BiGG integration tool workflow implemented in *merlin*. Users have access to BIT within *merlin*’s graphical interface. The genome sequence fasta file is submitted to the BIT webserver for an organism of interest. A bidirectional BLAST similarity search is performed against the protein sequences available at the BiGG models database (1). This process allows the identification of homologous genes in the database. Then, Gene-Protein-Reaction (GPR) associations are elaborated based on the homologous genes and reactions available in the BiGG models database (2). Identified reactions are further integrated and validated based on the associated metabolites, namely reactants and products (3). Finally, the model can be exported or integrated into *merlin*’s workspace.

As shown in the first step of Figure 1, BIT performs bidirectional BLAST similarity searches between the organism’s genome sequences and protein sequences available at the BiGG Models database. As a result, reciprocal matches between the direct and reverse homology searches allow identifying homologous genes between the organism of interest and the BiGG Models database.

BIT performs the association between the homologous genes found in the database and corresponding BiGG reactions in the next step. As described in stage 2 of Figure 1, GPR associations are used to find the BiGG reactions to be included in the draft metabolic network.

**Figure 2:**
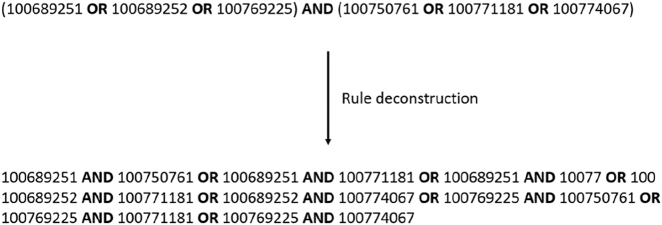
Decomposition of the GPR association assigned to the PI45P3K_cho reaction in BiGG models database.

For that, GPR associations are decomposed in a set of Boolean logic rules using the common operators “AND” and “OR”. Then, the integration of BiGG reactions in the draft reconstruction is based on the evaluation of the Boolean logic expression using the homologous genes and similarity searches. The assessment of each Boolean logic rule is performed according to the following principles:

(i) When the GPR association contains a single gene, the corresponding homologous gene must be present in the similarity searches to consider the BiGG reaction in the draft model.
(ii) When the GPR association only contains “OR” operators, a single homologous gene present in the similarity searches is enough to assemble a partial GPR association and consider the BiGG reaction into the draft model.
(iii) When the GPR association only contains “AND” operators, all homologous genes must be present in the similarity searches to assemble a complete GPR association and consider the BiGG reaction into the draft model.
(iv) When the GPR association contains both “OR” and “AND” operators, the Boolean logic rule is decomposed by the “OR” operator and evaluated as in the principle (ii). As demonstrated in the example of Figure 2, the GPR association of the PI45P3K_cho reaction available in the BiGG Models database can be simplified by the “OR” operators into a single Boolean logic expression.

Once all GPR associations have been evaluated using the homologous genes, all identified reactions are integrated and validated to avoid redundancy (stage 3 in Figure 1). As the BiGG Models database contains duplicated reactions due to the reactions’ reversibility, models’ compartmentalisation or compounds’ protonation state, BIT would automatically integrate all reactions in the draft metabolic network. Despite increasing the redundancy of reactions in the draft model, these cases are resolved by verifying that the relevant intervenient metabolites are the same in the redundant reactions.

After integrating the BiGG reactions, BIT assembles a draft metabolic network that can be exported as an SBML model [18]. Alternatively, the draft model can be integrated into the *merlin* workspace to be curated and refined.

## 3 Methods

### 3.1 Genomes’ Comparative Functional Analysis

The protein sequences of the organisms present in BiGG models and three case study bacteria were retrieved from NCBI’s RefSeq database (*S. thermophilus –* NZ_LR822015; *X. fastidiosa –* NC_004556.1; *M. tuberculosis –* NC_000962.3). Recogniser (version 1.4.6) [19] was run on the protein sequences to compare the functional similarity between the organisms with the following parameters: threads set to 14, maximum e-value set to 1e-10 and Clusters of Orthologous Genes (COG) [20] set as the reference database. Only COG identifiers belonging to the “METABOLISM” general functional category were selected for further analyses.

A binary matrix was generated to perform a Principal Components Analysis (PCA) of functional characterisations, with rows representing organisms, columns representing the COG identifiers, and 0 and 1 representing the absence or presence of COGs, respectively. Variables with low variance were removed, and standardisation was applied using the VarianceThreshold and StandardScaler methods of the scikit-learn package [21]. Plots were generated with the PCA method of the scikit-learn package, comparing functional characterisation at the Domain and Phylum levels (Figure 1).

### 3.2 Draft models

*S. thermophilus*, *X. fastidiosa*, and *M. tuberculosis* were the selected organisms for draft reconstruction with BIT and CarveMe [9]. For each organism, seven draft models were generated with BIT. Three different approaches were used, namely:

1. *all models templates*, which selects all the models from BiGG to build with available genome sequences;
2. *selected models template*, with models of the three metabolically closest organisms;
3. *random models templates*, in which five drafts were assembled, using three random models as templates in each model.

For estimating the closest organisms to *S. thermophilus*, *X. fastidiosa*, and *M. tuberculosis,* metabolic functions wise, results from Genomes’ Comparative Functional Analysis were used to create a distance matrix, where the presence/absence of COGs was quantified between all organisms and the top three selected.

Parameters for GEMs reconstruction with BIT were as follows: e-value, bit score and query coverage were set to 1e-20, 50 and 0.75, respectively, and BIT did not include BiGG reactions with missing or with incomplete GPRs. CarveMe was run with default parameters [9], without gapfilling.

### 3.3 Quantitative analysis of draft models

The diversity of reactions and functional annotation was analysed for the draft models. The COBRApy package [22] was used to read the draft models in SBML [18] format. The draft models were compared concerning their BiGG reaction and metabolite identifiers, and their COG annotations. To determine COG identifiers of each model, the RefSeq identifiers of the enzymes indicated in the GPRs associations of the model were retrieved, and cross-referenced with the annotation obtained in Section 3.1, in order to obtain the collection of COG identifiers of each model.

The first approach to compare reactions, metabolites and COG identifiers was Venn diagrams. This analysis aimed to assess how the different tools and templates affected the variability of the drafts GEM. The Venn diagrams were plotted using the matplotlib-venn package (available at https://github.com/konstantint/matplotlib-venn) for Python 3.8.

The draft models’ reactions and metabolites were further compared using PCA. A binary matrix was constructed, in which each row represented a draft model, and each column represented either a BiGG reaction identifiers, a BiGG metabolite identifiers or a COG identifiers, depending on the functional information being analysed. Matrix cells were filled with presence (1) or absence (0) of each identifier. Low-variance features (columns s^ 2 = 0) were removed using scikit-learn [21]. In addition, standardisation was also applied to each binary matrix, using the standard score (z-score: *Z* = (*x* − *μ*)/*σ*). A single PCA analysis was performed for each binary matrix using scikit-learn. Principal Components (PC) 1, and 2 were selected for each analysis. PCA scores were plotted and coloured according to the set of template models. Each dot was annotated with a model-specific identifier, using the first letter of the genus and the first three letters of the species’ specific name. As five random-template models were created using a different set of template models each time, these models are also numbered accordingly.

### 3.4 Model assessment

The draft models were assessed to published manually curated GEMs [15–17]. The comparison was applied to all models’ reactions, excluding exchange, sink, and demand reactions. A confusion matrix was generated for each model and used to calculate the evaluation metrics. Precision (*P*), recall (*R*), and F1 score (F1) were used for this evaluation and calculated as demonstrated in the following equations:

(1) $$P=\frac{\text{TP}}{\text{TP}+\text{FP}}$$

(2) $$R=\frac{\text{TP}}{\text{TP}+\text{FN}}$$

(3) $$\text{F}1=2\cdot \frac{P\cdot R}{P+R},$$

where *P* is the precision, *R* is the recall, F1 is the F1 score, TP refers to the true positives, FP to the false positives, and FN to the false negatives. Reactions present in both the reference model and the generated drafts were deemed TP. Moreover, reactions present in the drafts but missing in the reference model were regarded as FP. Conversely, the reactions of the reference model that were not in the drafts were considered FN.

According to these metrics, models with high R have an increased number of reactions correctly included (TP) and a low number of reactions missing (FN); thus, the efforts of manually curating the model are mostly associated with removing eventual FPs and adding few missing reactions (FN). On the other hand, models with low R have a high number of missing reactions (FN), requiring a more time-consuming refinement focused on adding the absent information.

Models with high P have an increased number of reactions correctly included (TP) when assessed to the number of reactions incorrectly added (FP). Proportionally, models with low P have a high number of reactions incorrectly assigned compared with reactions correctly inserted. In this regard, low P models are more suitable than low R from a curator’s point of view, as it is less tiresome to remove additional information than thoroughly searching for the missing information.

The F1 score combines the precision and recall of the classifier into a single metric, allowing a more balanced and direct evaluation of the draft models.

## 4 Results and discussion

BIT was used to reconstruct seven draft metabolic models for each bacterium: *M. tuberculosis*, *S. thermophilus* and *X. fastidiosa.* The models were created using three different approaches, namely using *all models*, *selected models* and *random models* as templates. CarveMe [9] was also used to develop a draft model for each bacterium. Then, all models were analysed and assessed regarding the variability of reactions and compared with curated models.

### 4.1 Genomes’ comparative functional analysis

**Figure 3:**
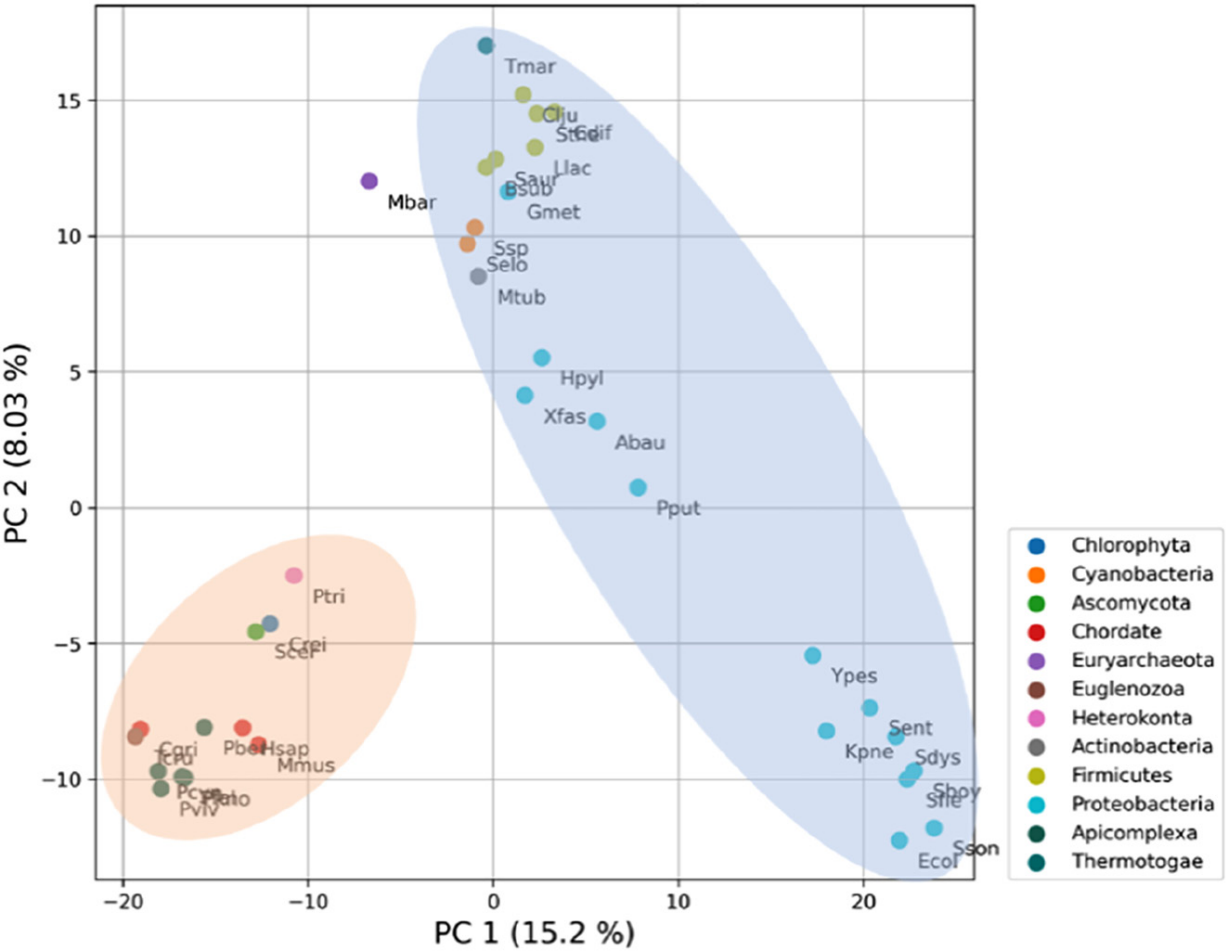
PCA plot of the COG-annotated metabolic functions obtained for all organisms present in BiGG database and for *S. thermophilus* and *X. fastidiosa*. The plot illustrates the contribution of principal components (PC) 1 and 2 to the variability of metabolic functions, including the percentage of explained variance. PCA scores are plotted, and the dots were coloured according to the organism’s phylum. The set of models for each organism is surrounded by ellipses that are only used for illustration purposes and do not represent real clusters. Eukaryotes and bacteria are represented by orange and blue ellipses, respectively, while the dot outside belongs to Archaea. An identifier for each organism was also used to annotate each dot, consisting of the first letter of the genus and the first three letters of the species’ second name.

The first step of this work was to analyse the metabolic variability of all species present in the BiGG database and *S. thermophilus* and *X. fastidiosa*, unavailable in BiGG. For this purpose, the recogniser tool [19] was used to retrieve the COG identifiers for all organisms and PCA was applied to check the variability of the COG-annotated metabolic functions. Figure 3 shows the contribution of principal components (PC) 1 and 2 to the variability of metabolic functions of all organisms. These components group BiGG organisms by phylum, meaning that organisms in the same phylum share more metabolic functions. In addition, Figure 3 shows an evident separation between eukaryotes and prokaryotes, which confirms the results published by the database authors in their latest publication [4]. Therefore, these results validate the use of metabolic COG identifiers to study the metabolic capacities of the organisms.

**Figure 4:**
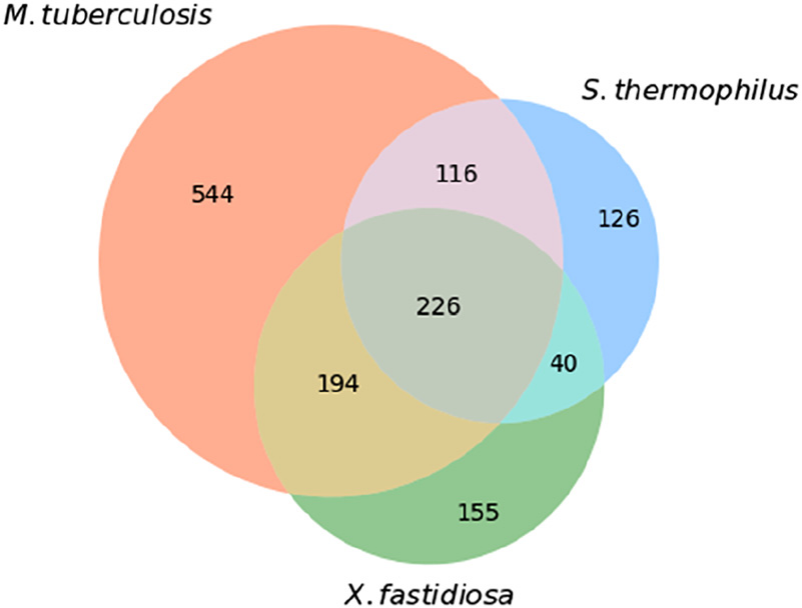
Venn diagram of the metabolic COGs obtained with recogniser for *M. tuberculosis, S. thermophilus* and *X. fastidiosa.* Venn diagrams show the number of unique COG identifiers shared between the three organisms.

The metabolic functions of the three case study bacteria were quantitively compared using the metabolic COG identifiers and a Venn diagram (Figure 4). Analysing Figure 4, 77% of all COGs identified for the three models are present in *M. tuberculosis,* of which 544 are unique for this organism, indicating that *M. tuberculosis* has more and more distinct metabolic functions. On the other hand, *S. thermophilus* and *X. fastidiosa* share most of their metabolic functions, having only 126 and 155 unique metabolic functions, respectively. Overall, the three bacteria only share 226 metabolic COGs, indicating that the respective models should reflect these metabolic differences. In addition, these microorganisms exhibit different features: *S. thermophilus* is a lactic acid Gram-positive bacterium; *X. fastidiosa* is a plant-pathogen Gram-negative; and *M. tuberculosis* is a well-studied bacterium and already has a GEM available on BiGG.

### 4.2 Model analysis

Draft metabolic models for the three case study bacteria were generated using BIT and CarveMe. The models used for the *selected models’ template*, were determined through similarity of metabolic functions, as shown in SI Table 1. The details of the 24 models generated for this study are summarised in SI Table 2.

**Figure 5:**
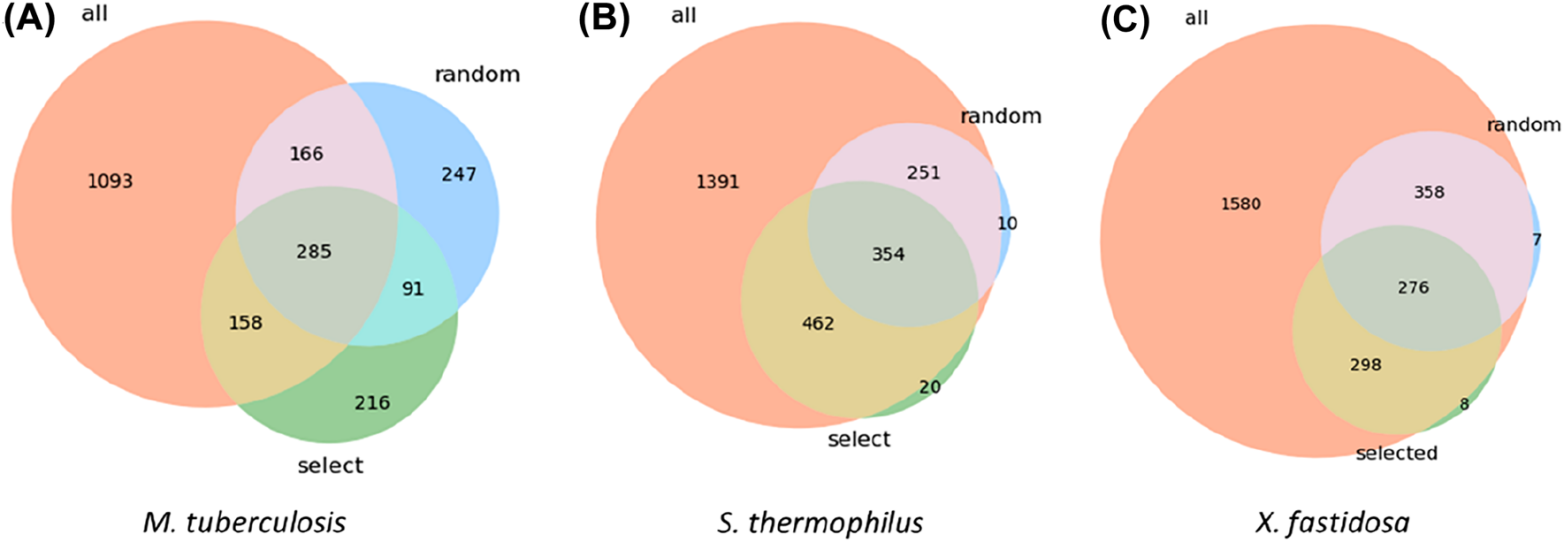
Venn diagrams for the reaction content of the different draft models for each organism. Draft models have been generated from BiGG using BIT with different approaches to building templates: *all models as templates*, *random models as templates* and *selected models as templates*. Venn diagrams show the number of reactions shared between the different models for *M. tuberculosis* (A), *S. thermophilus* (B), and *X. fastidiosa* (C).

The generated drafts were then compared to assess the effect of the template on the content of the models. For that, the reaction content was analysed in a Venn diagram (Figure 5), representing the number of unique and shared reactions between the different models of each bacterium.

As expected, *all models’ template* generated models with the highest number of reactions for all organisms. For *M. tuberculosis*, the model includes 75.4% of all reactions, of which 1093 are not shared with the models from other templates. This value is even greater for the other two bacteria: 1391 and 1580 unique reactions for *S. thermophilus* and *X. fastidiosa* models, respectively. Unsurprisingly, the *all models’ template* comprises most reactions available in the other templates. This is not the case of *M. tuberculosis*, as the BiGG database includes models for *M. tuberculosis*, which were used to reconstruct the model with the *all models’ template*, while in the other templates, homology searches may lead to different matches according to the models selected for the reconstruction. Therefore, these results suggest that the template used in BIT for reconstructing draft metabolic models impacts the reaction content of the generated models.

**Figure 6:**
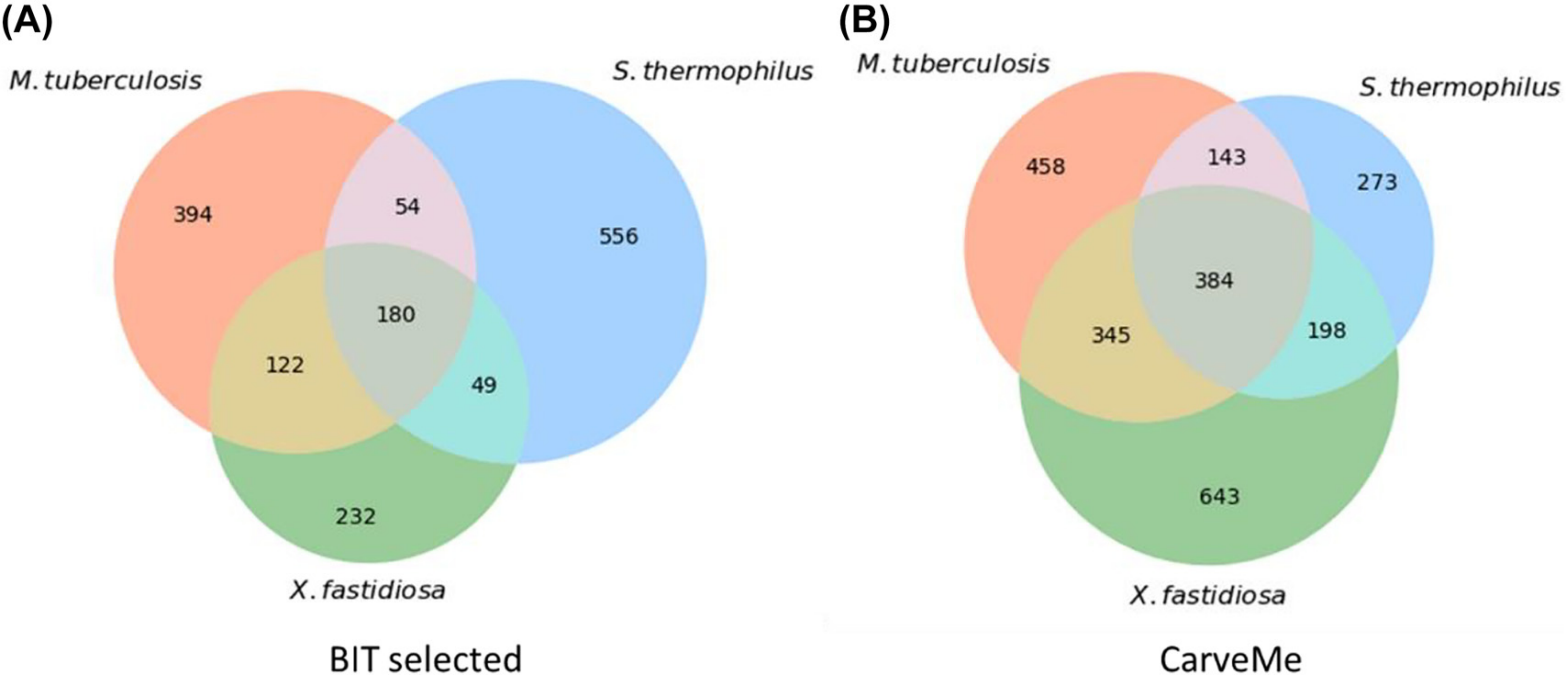
Venn diagrams of reactions from the draft models created with BIT’s selected template (A) and CarveMe (B), showing the number of reactions shared between the models of different organisms for the two approaches.

The draft models generated from BIT using the *selected* template and the models reconstructed by CarveMe were jointly analysed to investigate the differences in the models obtained with these two approaches. For this purpose, a Venn diagram was created for each method (Figure 6), representing the number of unique and shared reactions between the models of the three bacteria.

Regarding the models generated by BIT with the *selected models’ template*, 180 reactions are shared between the three models. Furthermore, the most distinct *selected* model belongs to *S. thermophilus*, with 556 unique reactions. In contrast, the model of *X. fastidiosa* is the one that presents more overlap with the other models, sharing 351 reactions, which represents 60% of its reaction content. However, the results do not agree with the inferences obtained from the metabolic annotation of the organisms (4.1), in which *M. tuberculosis* was the one with more unique COG-annotated metabolic functions (Figure 3).

On the other hand, the three models generated by CarveMe share 384 reactions, representing a slightly higher overlap than the models from BIT. In this case, the model with more unique reactions (643 reactions) belongs to *X. fastidiosa,* while the *S. thermophilus’* model is the most indistinctive, sharing 72.6% of its reaction content (725 reactions). These results are different from those obtained with BIT and from the results of the metabolic annotation (4.1).

CarveMe generates simulation-ready models, thus adding more reactions, such as sink and demand, to fill the model gaps. In contrast, BIT models require curation and gap-filling to be ready for simulation [9]. Therefore, although both approaches use BiGG to reconstruct draft metabolic models, the resulting drafts are considerably different.

The final step of the model analysis was to assess the metabolic functions included in the draft models. For that, genes included in the draft models were cross-referenced to the COG annotation of the genomes.

**Figure 7:**
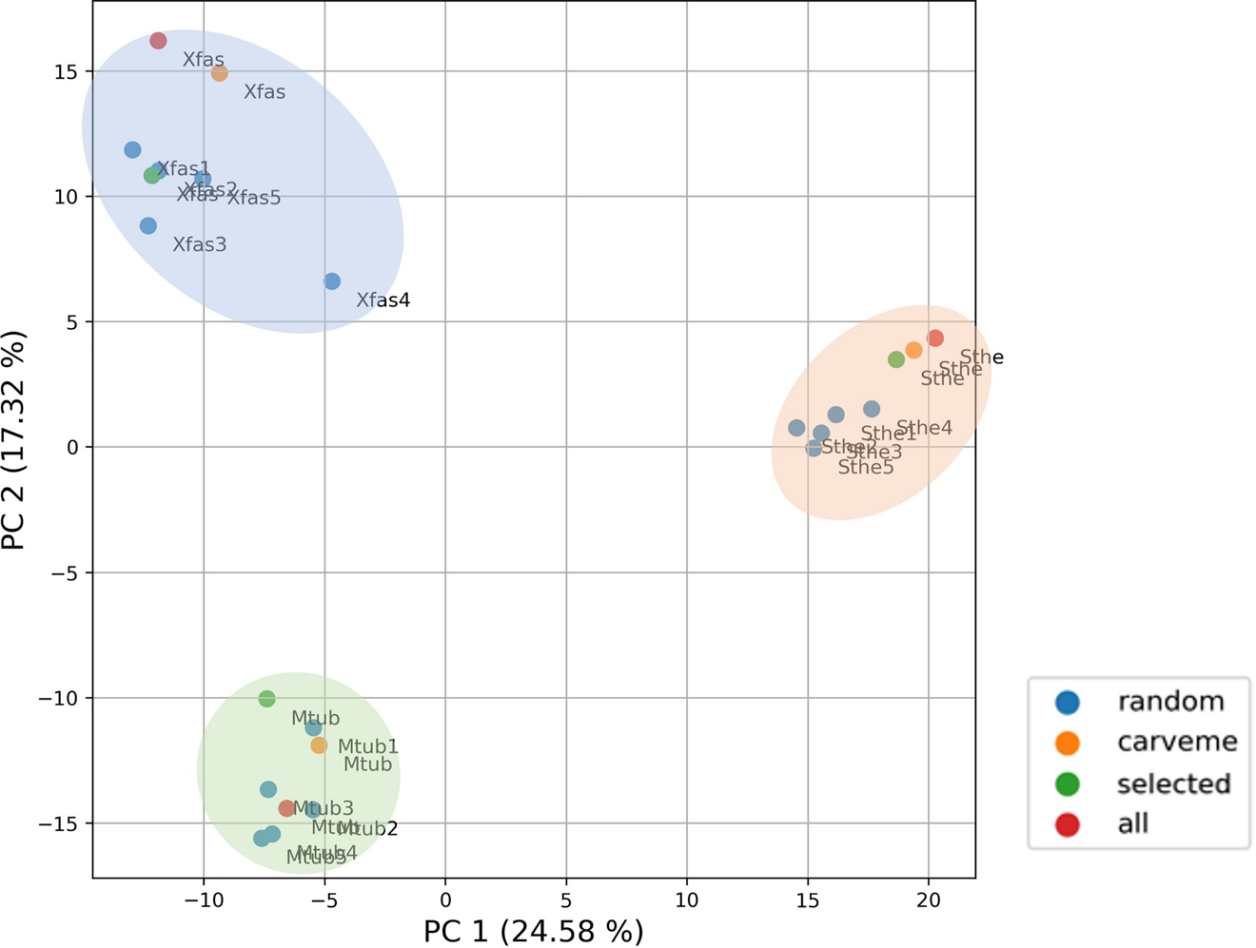
PCA plot of the COG-annotated metabolic functions of all draft models generated by BIT, using different templates, and by CarveMe, for each bacterium. The plot shows the influence of principal components (PC) 1 and 2 on the variability of metabolic features, including the percentage of variance explained by each PC. The dots represent each draft model, and their colour corresponds to the approach used in its reconstruction. Each dot is annotated with the organism-specific identifier, consisting of the first letter of the genus and the first three letters of the species’ second name. The set of models for each organism is surrounded by ellipses that are only used for illustration purposes and do not represent real clusters.

Hence, PCA was used to assess the variability of the COG metabolic functions present in the models for the three case study bacteria. The resulting PCA scores and the contribution of PC 1 and PC 2 to the variability of the data are shown in Figure 7. Analysing this figure, PC 1 unquestionably differentiates the metabolic functions of *S. thermophilus* models from those of *X. fastidiosa* and *M. tuberculosis* models. Similarly, PC 2 clearly separates the functional features of *M. tuberculosis* models from those of *X. fastidiosa* models*.*

These results show that the metabolic COG annotation of each model seems to be independent of the template or method used in the model reconstruction. All draft models from the same organism have a similar set of metabolic functions, different from other organisms’ models. Therefore, this suggests that the models’ metabolic functions are not influenced by the approach or the template, in contrast with the models’ reaction content analysis.

### 4.3 Models assessment

Twenty-four draft metabolic models were reconstructed from the BiGG database using BIT and CarveMe, and compared with manually curated models of the respective organisms under study. Herein, Tables 1 [Table j_jib-2022-0014_tab_002]–3 showcase the results of the mentioned comparison regarding P, R, and F1.

**Table 1:** Comparison of the evaluation metrics between draft models generated with CarveMe and with BIT for *M. tuberculosis*.

Model	Precision score	Recall score	F1 score
CarveMe	0.513	0.617	0.560
BIT all	**0.537**	**0.821**	**0.649**
BIT selected	0.396	0.269	0.320
BIT random 1	0.438	0.281	0.343
BIT random 2	0.401	0.259	0.314
BIT random 3	0.383	0.277	0.322
BIT random 4	0.383	0.323	0.350
BIT random 5	0.356	0.237	0.285

These bold values were included to highlight the model with the highest metrics.

**Table 2: j_jib-2022-0014_tab_002:** Comparison of the evaluation metrics between draft models generated with CarveMe and with BIT for *S. thermophilus*.

Model	Precision score	Recall score	F1 score
CarveMe	0.303	0.651	0.414
BIT all	0.155	**0.812**	0.261
BIT selected	0.406	0.724	0.520
BIT random 1	**0.508**	0.679	**0.581**
BIT random 2	0.413	0.478	0.443
BIT random 3	0.360	0.541	0.432
BIT random 4	0.466	0.703	0.560
BIT random 5	0.399	0.420	0.409

These bold values were included to highlight the model with the highest metrics.

**Table 3:** Comparison of the evaluation metrics between draft models generated with CarveMe and with BIT for *X. fastidiosa*.

Model	Precision score	Recall score	F1 score
CarveMe	0.378	0.615	0.468
BIT all	0.261	**0.672**	0.376
BIT selected	0.549	0.331	0.413
BIT random 1	0.590	0.570	0.580
BIT random 2	**0.652**	0.529	**0.584**
BIT random 3	0.585	0.387	0.466
BIT random 4	0.454	0.052	0.093
BIT random 5	0.500	0.381	0.432

These bold values were included to highlight the model with the highest metrics.

Table 1 shows the results of *M. tuberculosis* draft models. The best model in terms of P, R and F1 score was the *all models’ template* by a large margin. Accordingly, this draft model delivered a P of 0.537, an R of 0.821, and an F1 of 0.649, whereas the second-best model (CarveMe) presented 0.513, 0.617, and 0.560, respectively. Notably, the two best models significantly outperformed all *random* and *selected models templates*, especially considering R and F1. In this case, the second-best model (CarveMe) outperformed the best random and selected model templates by 29% and 21%, respectively.

There is a clear difference in performance between the draft models generated with the *all models’ template* or and CarveMe, which encompass a large part of the BiGG models’ database, and others that used a small part of it (*random* and *selected models’ templates*). This discrepancy might have occurred due to the utilisation of *M. tuberculosis* GEM to generate CarveMe and the *all* model, as it was stored in BiGG’s database. Such fact affected all other models’ performance, as they did not consider the mentioned GEM during reconstruction.

Nevertheless, the model generated with BIT’s *all models’ template* surpassed the model generated with CarveMe in all metrics. This result suggests that BIT can generate draft models with higher similarity to curated GEMs than CarveMe, particularly when the reconstruction is based on a curated GEM of the case study. Table 2 describes the assessment of all generated models of *S. thermophilus*.

The best-performance model regading R was generated using the *all models’ template* (0.812), followed by the model built using the *selected models’ template* with 0.724. The model generated with CarveMe was only the fifth-best model regarding R, having been outperformed by models generated with the *random* method of BIT. Models obtained with CarveMe and BIT *all models’ template* delivered a low number of FN and consequently a high R. This was most expected, as they were constructed considering a higher number of reactions (nearly all BiGG reactions), thus having lower chances of leaving reactions out, though with a higher chance of adding FPs to the model.

As for P, the top-performance model was the *random1* with 0.508, followed by *random4* (0.466) and *random2* (0.417). The models generated with BIT’s *all models’ template* and CarveMe obtained a P of 0.155 and 0.303, respectively. As expected, the number of FP in these models was considerably higher than the others, as these considered a higher number of reactions, having higher chances of adding “noise” to the model.

The models with the best F1 score were the *random1* (0.581), *random4* (0.560) and the *selected* (0.520), outperforming CarveMe, *random5*, and *all* models.

Table 3 showcases the performance of draft models of *X. fastidiosa.* In terms of R, the best models were the *all* with a performance of 0.672 and the one of CarveMe with 0.615. The former model surpasses the *random* and *selected* models by at least 10%, whereas the latter only performs better by a difference of 4.5%. As expected and similar to the models of *S. thermophilus*, the R values were higher for the models that made use of a broader collection of template models.

As before, models created with BIT’s *selected* and *random models templates* beat CarveMe and the *all models’ template* models’ performances in P. Accordingly, *random2* obtained the best P result (0.652), outperforming *random1* (0.590), *random3* (0.585), *selected* (0.549), *random5* (0.500), and *random4* (0.454).

Finally, the F1 top-performing models were unequivocally the *random2 models’ template* (0.584) and *random1 models’ template* (0.581). Interestingly, these two were also the best P and showed R values closer to CarveMe.

#### 4.3.1 General considerations

Eight models for each of the three organisms were reconstructed and compared against curated GEMs. The results obtained with these comparisons allowed us to reach a few conclusions.

As expected, generating models from template GEMs of the organisms under study is beneficial compared with not using those templates. Accordingly, the models of *M. tuberculosis* generated with CarveMe and the *all models’ template* method performed better for all metrics. Nevertheless, BIT’s *all models’ template* method surpassed CarveMe in all metrics.

R allows us to define how many TPs were predicted compared with the total number of real positives. In short, it evaluates how many reactions are left behind during reconstruction, as lower R represents that there are few TP compared with FN. From a curator’s point of view, it is easier to remove reactions than search and add new reactions to the model. Hence, a high recall indicates that the model’s curation process will be less tiresome and time-consuming. BIT’s *all* and *selected models’ template*, and CarveMe models were the best for this indicator. Notably, the two best BIT models obtained higher R than CarveMe.

High P translates in a low number of FP compared with all cases predicted as positive. Hence, high values of this metric indicate that a model has less “noise” and has more reactions in a potential curated model. On the other hand, a model with a high P in detriment of R would require more effort in the curation process, as there would be several reactions missing. Therefore, the best models of *S. thermophilus* and *X. fastidiosa* were generated with the *random models’ template* methods of BIT.

Finally, F1 combines P and R into a single metric, directly evaluating the performance of draft models’ construction in terms of balance between FP and FN. High F1 indicates that the reconstruction method is not leveraging errors, both adding and discarding reactions. The best models were generated using BIT random models’ template methods according to this definition.

## 5 Conclusions

This study concludes that BIT and CarveMe reconstruct different drafts for specific organisms using BiGG as a source of template models. Analysis of the present reactions and metabolic functions of the models’ genes revealed a clear distinction between the drafts reconstructed for each studied organism.

Even though the distribution of genes’ metabolic functions revealed small variability between the different templates used, the comparison of draft reactions found in each template showed that a careful selection of the template models is required as it impacts the reactions added to the draft.

Moreover, when comparing the reactions of the generated drafts to curated and validated models, BIT’s models provided higher metrics’ than CarveMe models. As for the best templates to use, it depends on the end goal. Suppose the aim is to have the lowest number possible of FNs. In that case, even if that means having a high number of FPs, the best templates seem to be the *all models’ template*, as removing reactions is less time consuming than adding missing reactions, and the user may not mind the extra “noise” in the draft. However, if the goal is to have the lowest number of FPs and FNs combined, the F1 metric suggests that the random template leads to better results.

Nevertheless, it is worth considering that BIT only retrieves an initial draft model for the organisms under study. Further curation and validations of the model are still required to obtain a final model that can be used for phenotype prediction. In addition, the conclusions reached by this work can only be stated when considering simple bacterial organisms. Due to the distribution of studied organisms, the applicability of BIT to eukaryotes or bacteria with a different metabolic profile was not assessed and cannot be guaranteed. Hence, expected future work includes extending this analysis to more organisms.

## Supplementary Material

Supplementary Material Details
