# Supplementary material for: Systematic assessment of template-based genome-scale metabolic models created with the BiGG Integration Tool
Source: J Integr Bioinform. 2022 Sep 5;19(3):20220014. doi: 10.1515/jib-2022-0014 (PMC9521827; doi:10.1515/jib-2022-0014)
Supplement: Supplementary file 1 — Supplementary Material Details [file j_jib-2022-0014_suppl.zip › JIB.2022.0014.R1/SI_Table_1.docx]

**Table 1 – Organisms from the BiGG database most metabolically similar to S. thermophilus, X. fastidiosa and M. tuberculosis. Number in parentheses represents the functional distance among the organisms, i.e., the number of COG IDs present in one and absent in the other.**

| S. thermophilus | X. fastidiosa | M. tuberculosis |
| --- | --- | --- |
| Lactococcus lactis subsp. cremoris (213) | Helicobacter pylori (378) | Synechocystis sp. (391) |
| Staphylococcus aureus subsp. aureus (362) | Acinetobacter baumannii (386) | Geobacter metallireducens (397) |
| Thermotoga maritima (387) | Synechococcus elongatus (399) | Synechococcus elongatus (400) |
