# Supplementary material for: Systematic assessment of template-based genome-scale metabolic models created with the BiGG Integration Tool
Source: J Integr Bioinform. 2022 Sep 5;19(3):20220014. doi: 10.1515/jib-2022-0014 (PMC9521827; doi:10.1515/jib-2022-0014)
Supplement: Supplementary file 1 — Supplementary Material Details [file j_jib-2022-0014_suppl.zip › JIB.2022.0014.R1/SI_Table_2.docx]

**Table 1: Description of the draft models generated from BIGG database using BIT with different templates and CarveME, for the three organisms, M. tuberculosis, S. thermophilus and X. fastidiosa.**

| **Organism** |  | Genes | Reactions | Metabolites |
| --- | --- | --- | --- | --- |
| *M. tuberculosis* | **all** | 909 | 1702 | 1985 |
|  | **selected** | 465 | 750 | 856 |
|  | **random1** | 460 | 714 | 829 |
|  | **random** | 412 | 737 | 877 |
|  | **random3** | 485 | 811 | 943 |
|  | **random4** | 512 | 934 | 1110 |
|  | **random5** | 447 | 748 | 962 |
|  | **CarveMe** | 944 | 1484 | 1087 |
| *S. thermophilus* | **all** | 491 | 2458 | 2633 |
|  | **selected** | 369 | 839 | 907 |
|  | **random1** | 305 | 632 | 803 |
|  | **random2** | 276 | 539 | 635 |
|  | **random3** | 293 | 702 | 860 |
|  | **random4** | 319 | 719 | 825 |
|  | **random5** | 293 | 494 | 611 |
|  | **CarveMe** | 518 | 1162 | 807 |
| *X.*  *fastidiosa* | **all** | 503 | 2513 | 2706 |
|  | **selected** | 329 | 583 | 694 |
|  | **random1** | 356 | 934 | 1238 |
|  | **random2** | 340 | 786 | 908 |
|  | **random3** | 300 | 639 | 847 |
|  | **random4** | 54 | 110 | 207 |
|  | **random5** | 313 | 739 | 926 |
|  | **CarveMe** | 557 | 1783 | 1270 |
