# Supplementary material for: Systematic assessment of template-based genome-scale metabolic models created with the BiGG Integration Tool
Source: J Integr Bioinform. 2022 Sep 5;19(3):20220014. doi: 10.1515/jib-2022-0014 (PMC9521827; doi:10.1515/jib-2022-0014)
Supplement: Supplementary file 1 — Supplementary Material Details [file j_jib-2022-0014_suppl.zip › JIB.2022.0014.R1/SI_Table_2.pdf]

***Table 1*: Description of the draft models generated from BIGG database using BIT with different templates and CarveME, for the three organisms, *M. tuberculosis, S. thermophilus* and *X. fastidiosa*.**

ORGANISM		GENES	REACTIONS	METABOLITES
M. tuberculosis	all	909	1702	1985
	selected	465	750	856
	random1	460	714	829
	random	412	737	877
	random3	485	811	943
	random4	512	934	1110
	random5	447	748	962
	CarveMe	944	1484	1087
S. thermophilus	all	491	2458	2633
	selected	369	839	907
	random1	305	632	803
	random2	276	539	635
	random3	293	702	860
	random4	319	719	825
	random5	293	494	611
	CarveMe	518	1162	807
X. fastidiosa	all	503	2513	2706
	selected	329	583	694
	random1	356	934	1238
	random2	340	786	908
	random3	300	639	847
	random4	54	110	207
	random5	313	739	926
	CarveMe	557	1783	1270
